# Potential Mechanisms Supporting the Value of Motor Cortex Stimulation to Treat Chronic Pain Syndromes

**DOI:** 10.3389/fnins.2016.00018

**Published:** 2016-02-11

**Authors:** Marcos F. DosSantos, Natália Ferreira, Rebecca L. Toback, Antônio C. Carvalho, Alexandre F. DaSilva

**Affiliations:** ^1^Universidade Federal do Rio de JaneiroRio de Janeiro, Brazil; ^2^Departamento de Radiologia, Faculdade de Medicina, Universidade Federal do Rio de JaneiroRio de Janeiro, Brazil; ^3^Headache and Orofacial Pain Effort, Department of Biologic and Materials Sciences and Michigan Center for Oral Health Research, School of Dentistry, University of MichiganAnn Arbor, MI, USA

**Keywords:** chronic pain, headache, migraine, motor cortex stimulation, neuromodulation, transcranial direct current stimulation, transcranial magnetic stimulation

## Abstract

Throughout the first years of the twenty-first century, neurotechnologies such as motor cortex stimulation (MCS), transcranial magnetic stimulation (TMS), and transcranial direct current stimulation (tDCS) have attracted scientific attention and been considered as potential tools to centrally modulate chronic pain, especially for those conditions more difficult to manage and refractory to all types of available pharmacological therapies. Interestingly, although the role of the motor cortex in pain has not been fully clarified, it is one of the cortical areas most commonly targeted by invasive and non-invasive neuromodulation technologies. Recent studies have provided significant advances concerning the establishment of the clinical effectiveness of primary MCS to treat different chronic pain syndromes. Concurrently, the neuromechanisms related to each method of primary motor cortex (M1) modulation have been unveiled. In this respect, the most consistent scientific evidence originates from MCS studies, which indicate the activation of top-down controls driven by M1 stimulation. This concept has also been applied to explain M1-TMS mechanisms. Nevertheless, activation of remote areas in the brain, including cortical and subcortical structures, has been reported with both invasive and non-invasive methods and the participation of major neurotransmitters (e.g., glutamate, GABA, and serotonin) as well as the release of endogenous opioids has been demonstrated. In this critical review, the putative mechanisms underlying the use of MCS to provide relief from chronic migraine and other types of chronic pain are discussed. Emphasis is placed on the most recent scientific evidence obtained from chronic pain research studies involving MCS and non-invasive neuromodulation methods (e.g., tDCS and TMS), which are analyzed comparatively.

## Introduction

Pain is clinically identified as an early and disabling symptom, extremely frequent and common to various diseases. However, rather than simply a sensory phenomenon, pain is better characterized as a complex experience extending beyond the sensory-discriminative component of pain, or the individual capacity to identify the nature (e.g., intensity, location, and duration) of a particular noxious stimuli. The affective-emotional aspect of pain (e.g., unpleasantness), as well the involvement of attention, memory of previous experiences, and anticipation, termed the cognitive-evaluative pain dimension, are fundamental pieces of this still challenging and complex puzzle (Melzack and Casey, 1968; Merskey et al., 1994; McMahon, 2013).

According to a widely applied definition, pain can be differentiated into either acute or chronic. Acute pain is produced by tissue injury and concurrent activation of local nociceptive transducers. Usually related to trauma, invasive procedures, or as a symptom occurring during the course of some pathological process, acute pain characteristically lasts for only a limited amount of time and resolves as soon as its primary source ceases. While chronic pain may also be initiated by local injury or disease, it usually persists for a longer period of time and tends to be maintained by factors not directly linked to the original event (Fishman et al., 2010). In fact, the International Association for the Study of Pain (IASP) defines chronic pain as pain experienced every day for 3 months over a period of 6 months (Merskey et al., 1994). Chronic pain is not only a clinical struggle but also a social burden, with enormous economic costs to healthcare systems across the globe (Patel et al., 2012). Due to its high prevalence (Verhaak et al., 1998; Elliott et al., 1999, 2002; Breivik et al., 2006) and deleterious impact on patients' quality of life (Patel et al., 2012), chronic pain receives considerable attention from both clinicians and researchers worldwide. Most of this attention is focused on better comprehending the multifaceted biological aspects of chronic pain and developing novel therapies that will permit more adequate relief from such an incapacitating condition. In this respect, recent years have seen an increased research interest in the study of different methods to modulate the activity of neurocircuits with the purpose of treating chronic pains. These methods include both surgical and non-invasive approaches, and their treatment effects have been studied alone and when combined with pharmacological therapies. While the clinical application of brain stimulation techniques dates back to the last century, the related technologies have evolved considerably as scientific evidence accumulated within the field (Kumar and Rizvi, 2014). Furthermore, the efficacy and reliability of different neuromodulatory methods, with stimulation delivered to distinct cortical/subcortical and even peripheral zones, have been tested in the treatment of several chronic pain disorders. Intriguingly, when retrospectively analyzing the scientific evidence accumulated throughout the last 25 years, the stimulation of motor cortical areas, mainly the primary motor cortex (M1), either non-invasively or by implanted electrodes has been consistently reported as an effective analgesic strategy to provide chronic pain relief, especially those of predominantly neuropathic origins (Tsubokawa et al., 1991a; Hosomi et al., 2013; Hagenacker et al., 2014; Ngernyam et al., 2015; Radic et al., 2015).

The advent of neuroimaging has allowed for the identification of an intricate network of brain structures that contributes to the pain experience and their specific roles in each dimension of the whole phenomenon. Most of those brain areas are multimodal, responding to both noxious and salient non-noxious stimuli (Mouraux et al., 2011). It has been recognized that this network includes the primary and secondary somatosensory cortices (SI and SII), the cingulate cortex, the posterior parietal cortex, and the pre-frontal cortex. Also taking part in this network are the thalamus, insula, and several brainstem structures, in addition to other interconnected brain areas. Not surprisingly, there is relatively scarce information regarding the contribution of the motor cortex to this process. Although the effects of pain on motor function have been well-documented, the participation of motor brain areas in the mechanisms that lead to chronic pain is still not completely understood (Farina et al., 2003). Therefore, one question remains unsolved: Why and how is motor cortex stimulation, in particular M1 stimulation, effective in treating chronic pain patients?

Based on scientific evidence currently available, this paper provides a critical review on the topic by exploring the putative mechanisms that explain the effectiveness of two methods of non-invasive neuromodulation, transcranial direct current stimulation (tDCS) and transcranial magnetic stimulation (TMS), when applied to the motor cortex for the treatment of chronic pains. To this purpose, the scientific evidence obtained with the invasive procedure, termed motor cortex stimulation (MCS), is always used as a reference.

## Are they effective? state-of-the-art non-invasive neuromodulatory technologies available to ameliorate chronic pain

Given the clinical challenges that chronic pain management presents, scientific pain researchers have directed their focus toward the development of novel technologies and enhancement of known strategies that permit the modulation of cortical excitability in humans through non-invasive or minimally invasive procedures. Over the past years, several studies have investigated the analgesic effects of epidural/subdural MCS, especially in refractory or intractable neuropathic pain (Meyerson et al., 1993; Tsubokawa et al., 1993; Nguyen et al., 1999, 2008, 2009; Saitoh et al., 2003; Nuti et al., 2005; Rasche et al., 2006; Velasco et al., 2008; Fontaine et al., 2009; Lefaucheur et al., 2009). Regarding non-invasive procedures, the first study demonstrating the analgesic effects of high-frequency rTMS of the motor cortex was performed in neuropathic pain patients (Lefaucheur et al., 2001). Later, the analgesic effects of anodal tDCS applied to the motor cortex was again reported in patients with neuropathic pain due to spinal cord injury (Fregni et al., 2006a) and also in fibromyalgia patients (Fregni et al., 2006b). In the following years, substantial data has emerged suggesting that distinct chronic migraine and pain syndromes can be successfully treated by tDCS (Antal et al., 2010, 2011; Mendonca et al., 2011; DaSilva et al., 2012; Jensen et al., 2013; Kim et al., 2013; Viganò et al., 2013; Villamar et al., 2013; Wrigley et al., 2013; Hagenacker et al., 2014; Schabrun et al., 2014; Bolognini et al., 2015; Donnell et al., 2015) and/or TMS (Lefaucheur et al., 2010b; Picarelli et al., 2010; Mhalla et al., 2011; Lee et al., 2012; Hosomi et al., 2013; Tzabazis et al., 2013; Hasan et al., 2014). Moreover, the value of rTMS to predict the long-term effects of MCS has been reported (Lefaucheur et al., 2004, 2011; André-Obadia et al., 2006, 2014; Hosomi et al., 2008).

Nevertheless, findings from systematic reviews of the methodology and results of studies investigating the role of non-invasive neuromodulation for pain control suggest that more clinical trials with rigorously designed protocols and larger samples sizes are still necessary to draw more accurate conclusions (Klein et al., 2015; Table 1). As reported in a recent meta-analysis, low or very low-quality evidence indicate that prefrontal low-frequency repetitive TMS (rTMS) is not effective for pain control, while a single dose of high-frequency motor cortex TMS provides short-term pain improvement. Conversely, according to an international group of experts, in cases of neuropathic pain the production of analgesic effects by high-frequency (≥5 Hz) rTMS of the motor cortex contralateral to the pain side has a level A of evidence (Lefaucheur et al., 2014). However, this statement cannot be extended to other stimulation settings, targets, or pain conditions. In addition, it is important to highlight the importance of long-term effects of rTMS protocols in pain therapy. Because of the short-lasting duration of the analgesic effects produced, it is still necessary to define and optimize maintenance protocols before considering rTMS as a valuable technique for the treatment of neuropathic pain in routine practice. So far, only a few studies have shown clinical improvement lasting several months from rTMS in patients with chronic pain syndromes (Mhalla et al., 2011; Hodaj et al., 2015).

**Table 1:** **Summary of the systematic reviews that investigated the methodology and results of studies exploring the effects of tDCS and TMS on pain management**.

	**Transcranial Magnetic Stimulation (TMS)**	**Transcranial Direct Current Stimulation (tDCS)**
Types of study	Systematic review and meta-analysis.	Systematic review and meta-analysis.
Quality of evidence	Low or very low-quality.	Low-quality.
Main findings	A single dose of high-frequency motor cortex TMS provides short-term pain improvement.	Not effective for chronic pain control (O'connell et al., 2014).
		Presence of scientific evidence indicating that anodal M1-tDCS significantly reduces pain levels in chronic pain patients (Vaseghi et al., 2014).
	HF rTMS and anodal M1-tDCS produce similar pain improvements and less adverse effects when compared to the FDA approved fibromyalgia pharmaceuticals.	
Limitations	High heterogeneity of the research protocols: differences with respect to the cortical targets for stimulation; differences in the number of stimulations per subject, with the presence of single and multiple-dose studies; application of low or high frequency stimulation (or differences in the current intensity); the type of pain disorder evaluated. Presence of adequate subject blinding during active and sham stimulation.	
Perspectives	The findings suggest that more clinical trials with rigorously designed protocols and larger sample sizes are still necessary to draw more accurate interpretations.	

Regarding tDCS, low-quality evidence does not yet suggest that it is effective for chronic pain control (O'connell et al., 2014). On the other hand, it is imperative to consider the high heterogeneity of the research protocols evaluated, including important differences with respect to the cortical targets chosen for stimulation [e.g., motor cortex and dorsolateral prefrontal cortex (DLPFC)]; differences in the number of stimulations per subject, with the presence of single and multiple-dose studies; application of low (≤1 Hz) or high frequency (≥5 Hz) stimulation, in the case of TMS; differences in the current intensity (usually 1 or 2 mA), in relation to tDCS; and of particular relevance, the type of pain disorder evaluated (e.g., nociceptive or neuropathic).

Indeed, chronic pain does not represent a single entity but a spectrum of disorders, triggered, and maintained by complex mechanisms (Basbaum et al., 2009). Therefore, it is possible to infer that TMS or tDCS could produce differential effects on each type of chronic pain disorder. For example, one systematic review focused on clinical research protocols that investigated the effects of low and high frequency (LF and HF, respectively) TMS and anodal tDCS (at intensities of 1 or 2 mA) in patients diagnosed with fibromyalgia. The review concluded that HF rTMS as well as anodal tDCS stimulation of M1 (M1-tDCS) offer similar pain improvements when compared to the FDA-approved fibromyalgia pharmaceuticals. The authors advocate that rTMS and tDCS should be considered when treating fibromyalgia patients, especially those individuals who are refractory to other (pharmacological) therapies or who do not tolerate their side effects (Marlow et al., 2013). Likewise, another meta-analysis supported that anodal M1-tDCS significantly reduces pain levels (represented by an average of nearly 15% pain reduction, measured with the visual analog scale—VAS of pain) in chronic pain patients (Vaseghi et al., 2014). Thus, despite the mounting evidence supporting the analgesic effects of non-invasive MCS, it is evident that additional clinical trials with standardized protocols and more robust data are needed to establish the extent to which tDCS and TMS can contribute to chronic pain management. Concurrently it is necessary to scrutinize the neurophysiological mechanisms as well as the neurochemical mediation associated with non-invasive brain stimulation.

## How do they act? putative mechanisms of non-invasive motor cortex stimulation

Despite the large number of studies exploring the clinical efficacy of non-invasive methods of neuromodulation, their neurophysiological fundaments are largely unknown and numerous uncertainties remain. For example, is it possible to revert ingrained neuroplastic changes with MCS? Do non-invasive methods of neuromodulation elicit a significant placebo effect? What scientific evidence has been obtained from basic sciences and neuroimaging studies and what does this evidence indicate? Although some of these questions have been at least partially addressed, one of the most elementary and intriguing questions persists: How does the stimulation of the motor cortex grant significant chronic pain relief?

An indication of one possible role of the motor cortex in pain arose many years ago when in 1971 a published report revealed cortical removals of both postcentral and precentral facial representations resulted in facial pain relief (White and Sweet, 1969; Lende et al., 1971). Yet, the role of the motor cortex only truly started receiving special attention from clinicians and researchers after Tsubokawa's work with MCS in 1991 (Tsubokawa et al., 1991a,b). Afterwards, this cortical region became a common target for neuromodulation when intended to treat pain (Meyerson et al., 1993; Nguyen et al., 1997; García-Larrea et al., 1999; Saitoh et al., 2000, 2003). Interestingly, a study using navigation-guided rTMS examined if significant pain improvement could also be achieved by stimulating cortical areas other than the precentral gyrus (M1) in patients with intractable deafferentation pain (Hirayama et al., 2006). Specifically, the other areas evaluated were the postcentral gyrus, the supplementary motor area and the premotor cortex (Hirayama et al., 2006). Confirming previous works, results of the study found that M1 stimulation produced significant pain relief. Conversely, stimulation of the adjacent areas was not effective in the cohort evaluated, corroborating the prominent role of the primary motor cortex in pain relief, and more precisely the importance of stimulation over the anterior bank of the central sulcus for pain treatment. Similarly, an experimental study involving healthy subjects who volunteered to receive capsaicin application reported significantly higher analgesic effects of rTMS over M1 when compared to the stimulation of the DLPFC and occipital cortex (Sacco et al., 2014). In fact, it has been described that, at least with MCS, optimal analgesic effects can be accomplished when the electrodes are positioned over the somatotopic representation (within M1) of the painful territory. To this purpose, it is mandatory to work on a detailed functional and anatomical mapping of the cortical representation of the painful zone prior to the stimulation (Nguyen et al., 2011).

The neurobiological machinery activated when the motor cortex is stimulated is a matter of intense debate. The first studies investigating the mechanisms of MCS pointed to a decrease in chronic pain-induced thalamic hyperactivity related to the stimulation (Tsubokawa et al., 1991a, 1993), which led to the conclusion that antidromic modulation of thalamocortical pathways could play a role in the analgesia induced by M1 stimulation (Nguyen et al., 2011). In this regard, there are special features in the structural and functional organization of the motor cortex that determine the effects following its electrical stimulation (Amassian and Stewart, 2003). It seems that cathodal electrical stimulation applied directly to the motor cortex (MCS) is associated with a preferential activation of the interneurons that run parallel to the cortical surface and an indirect stimulation of the pyramidal tract, generating indirect waves (I-waves) at the spinal cord. On the other hand, anodal electrical cortical stimulation of the motor cortex would preferentially activate the pyramidal cell axons, represented by the fibers that run perpendicularly to the cortical surface, and thus result in a direct stimulation of the pyramidal tract, producing early direct waves (D-waves) (Amassian et al., 1987; Amassian and Stewart, 2003; Nguyen et al., 2011). It has been described that the activation of the axons that run parallel to the cortical surface and the indirect generation of I-waves, accomplished through cathodal precentral gyrus stimulation, optimizes MCS analgesic effects (Lefaucheur et al., 2010a; Nguyen et al., 2011). Studies have confirmed that the most effective MCS electrode configuration for pain control is the one that generates I-waves (Lefaucheur et al., 2010a). Such findings could indicate that that MCS acts though the activation of top-down controls associated with intracortical horizontal fibers, instead of direct stimulation of the pyramidal tract (Nguyen et al., 2011). The same fundament can be transposed to rTMS. Similar to cathodal electrical stimulation, rTMS produces I-waves, and significant pain decrease when its coil is positioned in an anteroposterior orientation, whereas D-waves are formed when its coil is positioned in a lateromedial orientation (André-Obadia et al., 2008; Lefaucheur et al., 2010a; Nguyen et al., 2011). It has been proposed that the activation of the fibers that run parallel to the cortical surface in the precentral gyrus would lead to both orthodromic activation of corticofugal pathways as well as antidromic activation of thalamocortical pathways. Thus, it would influence pathways and structures that are distant from the side of stimulation (Nguyen et al., 2011).

The general view that the analgesic effects observed with M1 stimulation derives from the activation of areas far beyond the cortical zone where the stimulus is applied has been confirmed by neuroimaging studies (García-Larrea et al., 1999; Peyron et al., 2007). Some of those studies proved the ability of MCS to activate adjacent outer brain areas (e.g., orbitofrontal cortex—OFC, DLPFC) as well as remote inner brain structures, such as the insula and anterior, middle and posterior cingulate cortex, the putamen, the thalamus, and portions of the brainstem, including the periaqueductal gray matter (PAG) and the pons (García-Larrea et al., 1999; Peyron et al., 2007). Other studies have proved that rTMS can also influence the activity of a network that comprises cortical areas (M1, S1, supplementary motor cortex, dorsal premotor cortex, cingulate cortex, and insula), as well as the thalamus and basal ganglia (Strafella et al., 2003; Bestmann et al., 2004). It is important to highlight that all of those aforementioned elements of the human brain are largely recognized by their direct or indirect involvement in pain processing (Peyron et al., 2000; Zubieta et al., 2001). Remarkably, M1-rTMS consistently interferes with the activity of brain areas related to the emotional aspects of pain, including the cingulate cortex and insula, which explains the effects of M1 stimulation on the affective-emotional dimension of pain (Passard et al., 2007; Picarelli et al., 2010).

Changes in motor cortex excitability have also been explored for the purpose of understanding the neurophysiological aftereffects of M1 stimulation. Single- and paired-pulse TMS paradigms are important tools to assess motor cortex excitability parameters, including the resting motor threshold (RMT), the motor evoked potential (MEP) amplitude, the intracortical inhibition (ICI), the intracortical facilitation (ICF), and the electromyographic cortical silent period (CSP) (Ziemann et al., 1996; Sanger et al., 2001). It has been described that non-invasive MCS, achieved by tDCS or TMS, is associated with both immediate and long-lasting changes in motor cortex excitability (Wassermann et al., 1998; Nitsche and Paulus, 2000, 2001; Schambra et al., 2003; Jung et al., 2008). Noteworthy, it has been shown that changes in cortical excitability elicited by rTMS differ in healthy subjects (Wu et al., 2000; Romero et al., 2002) and chronic pain patients (Lefaucheur et al., 2006), suggesting that rTMS effects depend on the degree of cortical excitability present before the period of stimulation (Lefaucheur et al., 2006). Furthermore, previous studies have documented both increased (Schambra et al., 2003) and decreased (Wassermann et al., 1998) motor cortex excitability in the M1 contralateral to the stimulated side, which possibly indicates a role of TMS in the modulation of interhemispheric connections (Schambra et al., 2003).

Surprisingly, similar results could not be replicated with M1-tDCS. There is also evidence that tDCS does not act on glutamatergic transcallosal neurons, though it does influence the activity of ipsilateral inhibitory interneurons that receive transcallossal projections and that mediate transcallosal inhibition (Lang et al., 2004).

The results just presented suggest the functional effects of tDCS have a higher specificity, even though neuroimaging and computational modeling studies indicate conventional tDCS montages generate widespread electrical current that flows throughout outer brain regions and deeper structures (Faria et al., 2011; DaSilva et al., 2012; Neuling et al., 2012; Antal et al., 2014). In fact, it has been supported that reinforcement of both anatomical selectivity (e.g., guiding the electrical current to specific targets in the brain) and functional selectivity (e.g., activity and input selectivity) are required to promote a rational advancement of tDCS research (Bikson et al., 2013). In order to enhance the anatomical specificity and possibly its effectiveness in pain control, novel high-definition (HD)-tDCS montages that use ring instead of large electrodes have been tailored (DaSilva et al., 2015). In addition, the evaluation of the electrical current distribution through computational models have permitted the development of HD-tDCS montages (e.g., 2 × 2-HD) with the purpose of targeting specific areas of the motor cortex (e.g., head and face homuncular region of M1), thus reproducing the MCS parameters and principles (DaSilva et al., 2015; Donnell et al., 2015). However, further studies are necessary to establish the clinical relevance of enhancing anatomical specificity for tDCS-induced analgesia.

In addition to the mechanisms previously reported, the neurochemical mediation associated with the clinical outcomes of different neuromodulatory techniques has just started to be unveiled. The involvement of the endogenous opioid system, one of the most prominent analgesic mechanisms and target of the majority of opiates in this whole process, was initially indicated by a study that reported increased release of endogenous opioids in different pain-related brain areas after MCS (Maarrawi et al., 2007). Furthermore, it has been verified that the density of opioid receptor binding in the brain can predict the postoperative pain relief obtained with MCS in chronic pain patients (Maarrawi et al., 2013). Similarly, significant endogenous opioid release, confirmed by decreased binding potential of the selective μ-opioid receptor agonist [^11^C]carfentanil in pain-related regions (e.g., precuneus, PAG, prefrontal cortex, thalamus, anterior cingulate cortex, and insula), has been associated with a single session of anodal M1-tDCS in both healthy subjects (DosSantos et al., 2014) and in a single case of postherpetic neuralgia (DosSantos et al., 2012). These findings clearly indicate the contribution of the endogenous opioid system, most likely exerted through activation of the μ-opioid neurotransmission, in the analgesic effects induced by non-invasive stimulation of the motor cortex. Supporting this concept, a TMS study reported that intravenous administration of the opioid receptor antagonist naloxone significantly reduces the analgesia achieved by M1-rTMS. Remarkably, in that particular study naloxone administration did not impact the analgesic effects of rTMS when applied to the DLFC (de Andrade et al., 2011), suggesting that specific neuromechanisms can be elicited when distinct cortical regions are stimulated. Nevertheless, this conclusion needs to be explored in depth since another study found naloxone treatment performed prior to TMS resulted in a significant decrease of DLFC rTMS-induced analgesia (Taylor et al., 2012). It is important to emphasize that both naloxone studies were performed in healthy volunteers and the inclusion of chronic pain patients might have produced different findings.

It has also been postulated that mechanisms other than the activation of opioid receptors might contribute to the pain relief observed with different methods of neuromodulation (Lefaucheur et al., 2006; Nguyen et al., 2011; Foerster et al., 2015). Those mechanisms can be associated with the activation of inhibitory (GABAergic) as well as excitatory (glutamatergic) pathways. Remarkably, both pathways can be examined through the evaluation of some parameters of cortical excitability (e.g., ICI, ICF, and CSP) (Ziemann et al., 1996; Sanger et al., 2001). The scientific evidence currently available indicates that high frequency (10 Hz) rTMS can restore a defective ICI, which represents an impaired GABAergic neurotransmission present in chronic neuropathic pain patients (Lefaucheur et al., 2006). Moreover, according to the data available, the restoration of the defective ICI by rTMS correlates to the degree of pain relief (Lefaucheur et al., 2006).

One evidence that supports the involvement of the glutametergic neurotransmission in the analgesic effects driven by M1 stimulation is the focal release of dopamine in the putamen associated with M1-rTMS, an effect possibly induced by glutamatergic corticostriatal projections, originating in the stimulated motor cortex (Strafella et al., 2003). In fact, it has been described that the activation of descending mechanisms of pain control induced by M1 stimulation in experimental models of neuropathic pain presumably involves striatal dopamine D2 receptors (DRD2) (Viisanen et al., 2012). Additionally, it has been recently reported that the genetic regulation of DRD2 by 957C>T polymorphis affects the susceptibility for neuropathic pain and also pain modulation by rTMS (Jääskeläinen et al., 2014).

The participation of glutamate N-methyl-D-aspartate (NMDA) receptors in TMS-induced analgesia has also been explored. The establishment of this link has its origins in animal model studies (Ambriz-Tututi et al., 2012) and was confirmed in a study that showed a decrease in the analgesic effects induced by both M1 and DLPFC/PFC stimulation after the administration of the noncompetitive NMDA antagonist ketamine (Ciampi de Andrade et al., 2014). Such findings also point to the association between rTMS-induced analgesia and long-term potentiation- or long-term depression-like mechanisms, since NMDA exerts predominant control over synaptic plasticity and memory (Tsien, 2000; Li and Tsien, 2009). NMDA receptors could also be associated with tDCS-induced neuroplasticity (Liebetanz et al., 2002). The presence of long-term analgesic effects induced by rTMS (Lefaucheur et al., 2004) and its dependence on the frequency of stimulation (André-Obadia et al., 2006) support the presence of neuroplastic changes associated with rTMS. Indeed, the dependence on the frequency of stimulation used to induce synaptic plasticity and the duration exceeding the stimulation period, are characteristics of long-term potentiation and long-term depression (Cooke and Bliss, 2006). The ability of the NMDA-receptor antagonist dextromethorphan (DMO) to suppress the effects of both anodal and cathodal tDCS on cortical excitability also supports the contribution of NMDA receptors and synaptic plasticity to the tDCS effects (Liebetanz et al., 2002).

The results of clinical and experimental studies point to the participation of GABAergic mechanisms in the analgesia associated with MCS and M1-TMS (Bestmann et al., 2004; Lucas et al., 2011; Pagano et al., 2012; Cha et al., 2013). It has been proposed that such effect could be related to the thalamic modulation produced by M1 stimulation, which would act through GABA neurotransmission (Moisset et al., 2015). Moreover, the participation of the reticular formation components and monoaminergic projections in the analgesia induced by M1 stimulation has been examined. There is evidence from experimental models of neuropathic pain that the antinociception induced by the electrical stimulation of M1 possibly involves the rostroventromedial medulla as well as descending serotoninergic pathways (Viisanen and Pertovaara, 2010b). On the other hand, it has been reported that coeruleospinal noradrenergic pathways are not essential for this process (Viisanen and Pertovaara, 2010a). Nevertheless, considering the still limited scientific evidence, further studies will be necessary to expand the current knowledge regarding the neurotransmitters involved in MCS and M1 tDCS.

Recently, studies have also been explored the possible neurochemical actions of tDCS. Proton magnetic resonance spectroscopy (^1^H-MRS) studies demonstrated increases in Glx, a combined marker of glutamine and glutamate, and N-acetylaspartate (NAA), which is considered to be a measure of neuronal integrity, in the parietal cortex underneath the anode (Clark et al., 2011). Another study reported a significant decrease in Glx levels in the anterior cingulate cortex, related to active M1-tDCS (when compared to sham stimulation), in a cohort of fibromyalgia patients. There was also a trend toward an increase of GABA levels in the anterior insula when comparing active tDCS to baseline. Interestingly, the same study found a significant increase in NAA in the posterior insula when comparing sham tDCS vs. baseline, suggesting the presence of a placebo effect associated with M1-tDCS (Foerster et al., 2015).

**Figure 1:**
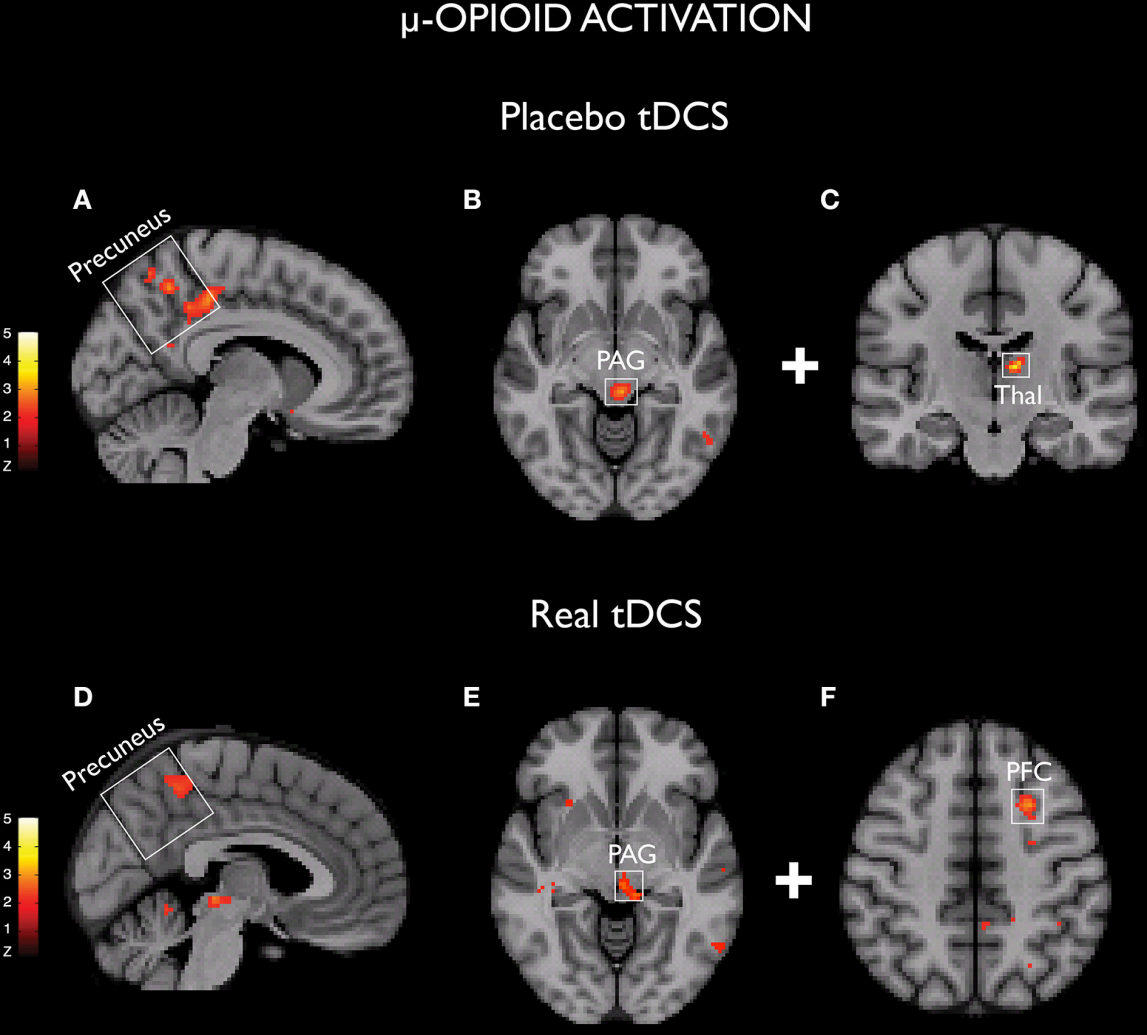
**Activation of μ-opioid receptors demonstrated with both sham (A–C) and real (D–F) tDCS (DosSantos et al., 2014)**.

Placebo is a factor that must always be considered when analyzing the effects of chronic pain therapies. Although several clinical trials involving non-invasive brain stimulation for pain relief have found significant differences between active and sham stimulation (Fregni et al., 2006a,b; Lee et al., 2012), considering the major role of the placebo effect for analgesia (Zubieta and Stohler, 2009) it is certainly possible that placebo might also play a role in the benefits of M1 stimulation for chronic pain treatment. This hypothesis has been recently evaluated with TMS (André-Obadia et al., 2011). The results suggest that the relative timing of sham and active TMS is an important factor to the placebo effect driven by this method. It has been demonstrated that placebo rTMS produces significant analgesia when applied after a successful active TMS session. Nevertheless, when following an unsuccessful active TMS session, placebo TMS tends to worsen pain. Interestingly, pain scores remained unaltered only when placebo TMS was applied before an active TMS session. Taken together, those results could reflect an unconscious conditioned learning related to placebo TMS. Regarding tDCS, considering that conventional montages produce widespread electrical current flow, a reasonable hypothesis that has emerged is that tDCS could reinforce the same brain networks that are usually activated by the expectations of clinical improvements (Schambra et al., 2014). This hypothesis would provide an alternative explanation for the beneficial effects observed with tDCS in depression studies, especially when a concurrent training (e.g., cognitive behavioral therapy) was not adopted and therefore activity-specificity was absent (Brunoni et al., 2012, 2013). In a recent study, we were able to demonstrate the presence of changes in the μ-opioid neurotransmission during both active and sham tDCS in humans. Surprisingly, we found concurrently (e.g., precuneus and PAG) as well as unrelated (e.g., PFC in active tDCS and thalamus during sham stimulation) μ-opioid activation (Figure 1), indicating that both shared and dissimilar mechanisms can drive the effects of sham and active tDCS in human subjects (DosSantos et al., 2014). These findings support the view that an earlier sham stimulation can build-up the effects of a subsequent active stimulation (DosSantos et al., 2014) and that heightening patients expectations with a placebo prior to active stimulation should also be considered (Schambra et al., 2014).

## Concluding remarks

Since the serendipitous observation that M1 stimulation produces significant clinical improvements in chronic neuropathic pain patients, this cortical region became the main target of several neuromodulatory techniques devoted to ameliorating chronic pain in human subjects. In fact, it has been reported that the stimulation of cortical regions adjacent to the primary motor cortex fail to produce similar analgesic effects, confirming the prominent role of M1 stimulation for pain control. Nevertheless, the intricate neurophysiological mechanisms that explain the clinical efficacy of M1 stimulation for pain relief are not completely understood. Evidence from MCS studies indicates that its analgesic mechanisms involve the activation of top-down controls related to the excitation of intracortical horizontal fibers. This concept can also be applied to TMS. However, results of neuroimaging studies also suggest that MCS and TMS act through modulation of deeper and remote brain structures related to pain, such as the insula, anterior, cingulate cortex, basal ganglia, thalamus, and brainstem. Interestingly, enhanced current flow in the same areas has also been demonstrated with tDCS. In addition, the neurochemical mediation driven by M1 stimulation has been recently unveiled in studies involving MCS, TMS, and tDCS. Opioidergic, glutamatergic, GABAergic and serotoninergic neurotransmissions are now considered components for the whole process. Nevertheless, there are still questions that must be answered, including those regarding the participation of other mechanisms of endogenous pain control, the clinical relevance of increasing anatomical and functional specificity in non-invasive procedures, and the presence and significance of a placebo effect. The answers to these questions are expected to be among the future perspectives of the field.

## Author contributions

MD, NF, RT, AC and AD drafted the manuscript. All authors read and approved the current version of this article.

### Conflict of interest statement

The authors declare that the research was conducted in the absence of any commercial or financial relationships that could be construed as a potential conflict of interest.
